# Performance Analysis and Admixture Optimization of GBFS-HPMC/Fiber Pervious Concrete

**DOI:** 10.3390/ma16196455

**Published:** 2023-09-28

**Authors:** Xiwen Yan, Xuezhi Wang, Chuanwu Sun, Ming Xin, Jingjing He

**Affiliations:** 1 School of Civil and Architectural Engineering, Liaoning University of Technology, Jinzhou 121001, China; 13880386103@163.com (X.Y.); scw18914870102@163.com (C.S.); xmmyemail@163.com (M.X.); 2 Power China Northwest Engineering Corporation Limited, Xi’an 710065, China; hejing_86@126.com

**Keywords:** granulated blast-furnace slag, hydroxyl propyl methyl cellulose, polypropylene plastic textile fiber, pervious concrete, gray correlation analysis

## Abstract

Permeable pavements can decrease the volume of stormwater, thereby mitigating the risk of flooding and reducing the urban heat island effect. This study investigated the influence of incorporating granulated blast-furnace slag (GBFS), hydroxypropyl methylcellulose (HPMC), and polypropylene plastic textile fiber (PPTF) on the mechanical properties and water permeability of pervious concrete. Orthogonal tests were employed to conduct the analysis. The findings indicate that the pervious concrete with GBFS, HPMC, and PPTF (termed GBFS-HPMC/fiber pervious concrete) exhibited the highest cubic compressive strength, ultimate tensile strength, and flexural strength. These values were 25.22 MPa, 3.36 MPa, and 5.39 MPa, respectively. The standard deviations for cubic compressive strength, split tensile strength, flexural strength, water permeability coefficient, and porosity, as calculated using SPSS, were 1.57, 0.1, 1.17, 0.35, and 0.4, respectively. Scanning electron microscopy (SEM) and energy-dispersive spectroscopy (EDS) were used to analyze the microstructure and compositional combinations of the pervious concrete. The analyses revealed that the calcium-silicate-hydrate (C-S-H) gel, produced by GBFS hydration, enhanced the bonding within the interfacial transition zone (ITZ) and between the fibers and aggregates. Additionally, the anchoring and supporting effects of the PPTF in the matrix contributed to stabilizing the overall matrix structure. Lastly, a gray correlation analysis was applied to optimize the admixture. The findings indicate that following the optimization, the cubic compressive strength increased by 7.2%, splitting tensile strength by 2.1%, and flexural strength by 2.5%. In summary, the mechanical properties of pervious concrete improved after optimizing the admixture.

## 1. Introduction

In recent years, the expansion of urban regions and the concentration of populations has resulted in the reduction of vegetated areas and natural habitats due to the construction of residential buildings and traditional concrete pavements. This situation hinders the natural infiltration of rainwater into the soil. During intense precipitation episodes, drainage systems cannot expel water rapidly, leading to waterlogging events in low-lying urban zones [1,2,3]. Concurrently, the absence of adequate stormwater runoff channels, combined with the incomplete treatment of stormwater and codischarge of domestic sewage, contributes to the contamination of freshwater resources [4,5]. Additionally, impervious surfaces reflect, retain, and emit a significant amount of solar radiation, contributing to the phenomenon known as the “urban heat island effect” [6,7].

Pervious concrete serves as a sustainable paving material that effectively addresses the shortcomings of traditional concrete pavements. This material consists of a mortar layer enveloped by coarse aggregate, which is bonded to form a porous, honeycomb-like structure. This structure offers the advantages of both water permeability and acoustic noise reduction [8,9,10]. The utilization of permeable pavement construction in “sponge cities” serves a vital role in absorbing rainwater, storing it, purifying it, and offering other capabilities on urban roads. This effectively helps in mitigating the decrease in groundwater levels and the occurrence of “waterlogging disasters” [11,12,13]. Additionally, pervious concrete pavement improves heat dissipation, reduces surface temperatures, and weakens the reflective strength of the road surface to solar radiation, offering a viable solution to the issue of high temperatures in urban areas [14,15]. Because of its porous nature, the strength of pervious concrete is not comparable to that of traditional concrete, limiting its use to sidewalks, plazas, parking lots, etc., with light loads. Currently, the focus of research is on improving the mechanical properties of pervious concrete while ensuring its water permeability.

With the promotion of China’s “dual-carbon” initiative, reducing carbon emissions to address climate change is the focal point of achieving overall energy-efficient and environmentally friendly development [16]. As a key material in the construction industry, cement releases carbon dioxide during its production and use, and the cement industry accounts for 7% of total global carbon emissions [17]. To promote the reduction of carbon emissions, mineral admixtures can be employed in engineering to partially substitute cement and decrease its usage. Examples of such admixtures encompass fly ash, silica fume, and slag micropowder.

Slag can induce the “volcanic ash effect”, reacting with calcium hydroxide produced by cement hydration to form hydrated calcium silicate [18], hydrated aluminum silicate, and other cementitious materials. This reduces the aggregation of calcium hydroxide crystals in the interfacial zone of the ITZ, thereby improving the adhesion between cementitious materials and aggregates [19,20]. Cement hydration generates significant heat, but slag can mitigate this heat, curbing the early contraction and deformation of pervious concrete, as well as cracks. Studies by Huang et al. revealed that the introduction of slag led to the formation of a new crystalline phase (new crystalline phases dominated by Na-Si-Al-H gels), resulting in enhanced surface integrity and notable mechanical improvements in pervious concrete [21]. Slag also offers a “gelling effect”, where the dicalcium silicate and calcium silicate present in the slag directly hydrate to produce water-hardening hydration products, enhancing coagulation and hardening for improved strength [22]. The unreacted slag is uniformly distributed throughout the pores and colloids, contributing to a “filling effect” [23].

The preparation of pervious concrete necessitates less gelling material, resulting in weaker bonding. However, the inclusion of hydroxypropyl methylcellulose can extend the mixing and forming time, enhancing the cohesion of the mix to prevent segregation. This compound boasts exceptional thickening and water retention properties, effectively enhancing the bond between the gelling material and aggregates, thus improving mechanical properties [24]. When cellulose is mixed with slag during concrete preparation, it significantly reduces shrinkage and inhibits cracking [25]. During the mixing process, cellulose reacts with water, forming molecular bonds that create a frozen film enveloping cementitious materials and aggregates, filling pores alongside the C-S-H gel to enhance structural compactness [26]. However, the air-entraining property of cellulose tends to introduce air bubbles during the mixing of permeable concrete, and excessive cellulose can result in closed pores, ultimately reducing the mechanical properties [27].

Incorporating fibers can reduce the high brittleness and poor resistance to bending and deformation inherent in pervious concrete, thus, enhancing its mechanical properties. Polypropylene plastic textile fibers, known for their ease of dispersion, acid and alkali resistance, and high toughness, are a suitable reinforcement for pervious concrete, effectively inhibiting crack formation and propagation [28,29,30]. Research indicates that the addition of plastisol fibers to concrete enhances its strength and toughness, leading to increased ultimate stress and ultimate strain [31].

Significant advancements have been made in the research of pervious concrete, both at the domestic and international levels. However, the constrained strength of pervious concrete limits its range of applications. Consequently, the current research focus centered on enhancing its mechanical properties while simultaneously considering the water permeability performance. Unlike previous studies that primarily examined individual materials, this paper presents a novel approach. This study devised a three-tier, four-variable L9 (3^4^) orthogonal experiment with the objective of exploring the influence of the slag replacement rate, cellulose additive, and PPTF additive on the mechanical and water permeability properties of pervious concrete. The study examined the compressive strength, tensile strength, flexural strength, permeability coefficient, and porosity of GBFS-HPMC/fiber-reinforced concrete. Gray correlation analysis and principal component analysis were employed to determine the optimal GBFS replacement rate, HPMC admixture, and PPTF admixture to enhance the mechanical properties of GBFS-HPMC/fiber-reinforced concrete. To promote the usage of GBFS-HPMC/fiber pervious concrete in real-world projects, it is essential to provide a reference.

## 2. Materials and Methods

### 2.1. Raw Materials

Silicate cement is primarily calcium silicate based, containing less than 5% limestone or granulated blast-furnace slag. It also incorporates an appropriate quantity of gypsum, which is crucial for creating water-hardened cementitious materials. The specific type of cement used in this context was P-O42.5, adhering to the standard silicate cement grade. It exhibits a density of 3.1 g/cm^3^ and is produced by the Bohai Cement Company Limited, located in Jinzhou City, China. Detailed specifications can be found in Table 1. The selection of this particular cement aligns with the guidelines outlined in GB175-2007, “Common Portland cement” [32]. The coarse aggregate comprises natural materials within the 5–10 mm range, sourced locally in Jinzhou City, China, with a mud content of 0.38% (Jinzhou, China). Ordinary river sand obtained locally in Jinzhou City, China, was utilized as the fine aggregate, constituting approximately 5% of the mixture. The SR eco-concrete reinforcement agent was provided by Jiangsu Jiajing Ecological Engineering Technology (Huaian, China). Conventional tap water was employed throughout the study. The slag utilized in this context was S95 grade granulated blast-furnace slag (GBFS) micropowder, sourced from Gongyi Longze Water Purification Material Co. It offers a specific surface area of 429m^2^/kg, and its performance parameters can be found in Table 2 (Gongyi, China). The choice of this particular slag aligns with the specifications outlined in GB/T18046-2017, “Ground granulated blast-furnace slag used for cement, mortar and concrete” [33]. The polymer of choice was hydroxypropyl methylcellulose (HPMC) with a fineness exceeding 100 mesh and a viscosity measuring 200,000 MPa-s, manufactured by Jinzhou Fuqiang Fine Chemical Co. (Jinzhou, China). The polypropylene plastic textile fiber, sourced from China Taian Companion Fiber Co. (Taian, China), possessed a length of 20 mm, and its specific properties are detailed in Table 3.

### 2.2. Experimental Program

This experiment employed an orthogonal test to study three influential factors: GBFS substitution rate (A), HPMC dosage (B), and PPTF dosage (C). The GBFS substitution rate was 15%, 20%, and 25%; the HPMC dosage was 0.1%, 0.15%, and 0.2% of the cementitious material; and the PPTF dosage was 0.1%, 0.3%, and 0.5%. The orthogonal design of the test is shown in Table 3, generated using the L9 (3^4^) orthogonal table. The PPTF dosages were 1%, 0.3%, and 0.5%. Table 3 displays the orthogonal design of the experiment with three levels and four factors. The blank columns in Table 4 estimate the experimental errors. Mineral admixture was used for internal mixing. The mix ratios for the pervious concrete are provided in Table 5, which were selected with a water–cement ratio of 0.28 and a target porosity of 15%.

### 2.3. Specimen Preparation

**Figure 1:**
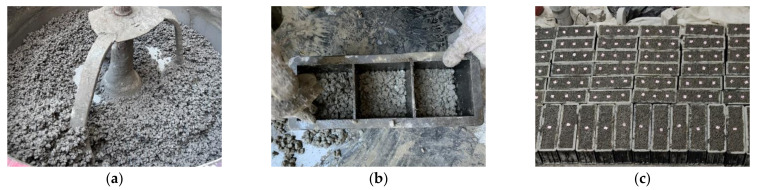
Specimen preparation and molding process: (**a**) specimen mixing; (**b**) insertion and pounding process; (**c**) specimen molding.

In the process of specimen preparation, first coarse aggregate, sand, PPTF, and part of the water were added and mixed for 30 s, then GBFS and cement were added and mixed for 60 s. Finally, HPMC reinforcement was added, followed by the addition of the remaining water, and the mixture was mixed for an additional 120 s. Subsequently, a combination of manual pounding and static compression molding techniques was employed. For each group of specimen molding, twelve specimens measuring 100 mm × 100 mm × 100 mm and three nonstandard specimens measuring 100 mm × 100 mm × 400 mm were prepared. In total, 150 specimens were prepared. Following the removal from molds, a total of 150 specimens were obtained, which encompassed 12 nonstandard specimens measuring 100 mm × 100 mm × 400 mm. These specimens were allowed to undergo natural conservation for a duration of 28 days, commencing from the 24th hour. The process of specimen preparation and molding is depicted in Figure 1.

### 2.4. Performance Test

Upon completion of the curing period, the cubic compressive strength, splitting tensile strength, and flexural strength were measured. A YAW-50000J compression–shear testing machine was used for loading. Tests for the porosity and water permeability coefficient were performed following the procedures outlined earlier.

#### 2.4.1. Cubic Compressive Strength

The specimen was placed at the center of the bearing plate on the press, and a continuous and uniform load was applied with a loading speed of 0.5 MPa/s. The calculation formula is provided in Equation (1). As per the guidelines specified in GB/T50081-2019, titled “Concrete physical and mechanical properties test method standard” [34], the test was conducted to determine the compressive strength of the cubic specimens.

(1) $$f_{c}=\frac{F}{A}\times \beta$$

where $f_{c}$ represents the cubic compressive strength (MPa), F represents the maximum load to which the specimen is subjected (N), A represents the area of the specimen to which the load is applied (mm^2^), and β represents the dimensional conversion factor of 0.95.

#### 2.4.2. Splitting Tensile Strength

The split compressive strength specimen was placed on the press support, and a continuous and stable load was applied at a rate of 0.015 MPa/s. The maximum load was recorded after the specimen fractured. The calculation formula is presented in Equation (2). In accordance with the standards outlined in GB/T50081-2019, “Concrete physical and mechanical properties test method standard” [34], the test was conducted to measure the splitting tensile strength of the specimens.

(2) $$f_{ts}=\frac{2P}{\pi A}\times \beta$$

where $f_{ts}$ represents the splitting tensile strength (MPa), P is the maximum load on the specimen (N), A is the loaded specimen’s area (mm^2^), and β is the size conversion factor of 0.85.

#### 2.4.3. Flexural Strength

The flexural strength specimen was placed on the press support and loaded at a rate of 0.05 MPa/s. The maximum load was recorded when the specimen fractured. The calculation formula is given in Equation (3). As specified in GB/T50081-2019, titled “Concrete physical and mechanical properties test method standard” [34], the test was carried out to determine the flexural strength of the specimens.

(3) $$f_{f}=\frac{Fl}{bh^{2}}\times \beta$$

where $f_{f}$ denotes the degree of flexural strength (MPa), F represents the maximum load on the specimen (N), l represents the distance between the two supports (mm), b represents the width of the specimen section (mm), h is the height of the specimen section (mm), and β denotes the size conversion factor of 0.85.

#### 2.4.4. Permeability Coefficient

**Figure 2:**
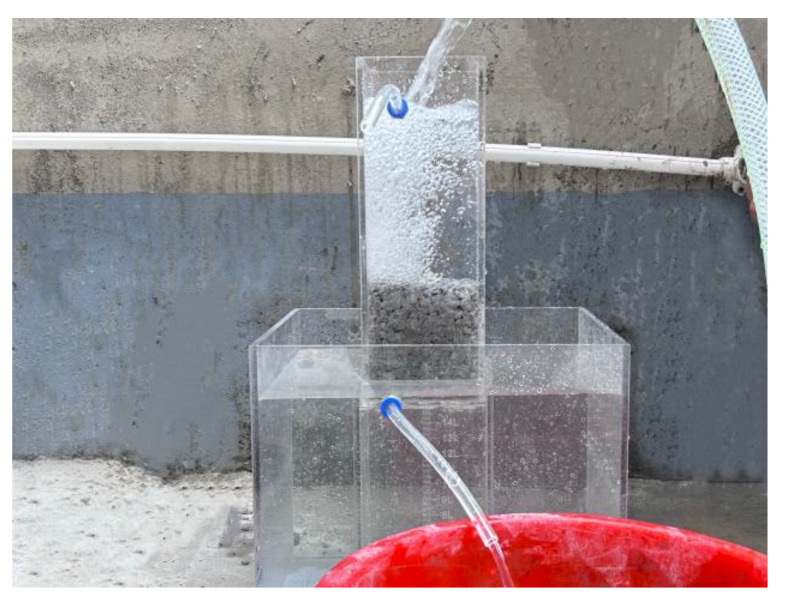
Permeability coefficient test equipment.

Once the specimen reached the age of 27 days, it was immersed in water for 24 h. After 28 days of aging, the permeability coefficient test was conducted. The calculation formula is presented in Equation (4), and the testing equipment for permeability coefficient is shown in Figure 2. In accordance with JCT2558-2020, “Pervious concrete”, and CJJ/T135-2009, “Technical specifications for pervious concrete pavement” [35,36], the permeability coefficient test was conducted using the fixed head method. The concrete permeability coefficient tester from Cangzhou Botronic Test Instrument Mall (Cangzhou, China) was employed for this purpose. To reach the appropriate age for the pervious concrete specimens, they were loaded into a prismatic device. The water supply valve was then opened, allowing water to enter the container. This process was carried out to maintain a specific water level in the prismatic device, approximately 150 mm. Once the device’s overflow port had a stable outflow of water, the amount of water that flowed out in 90 s was measured. This measurement was repeated three times for each specimen, and the average value was recorded.

(4) $$K_{T}=\frac{QL}{AHt}$$

where Q represents the amount of water flowing out in 90 s, t is taken as 90 s, A is the upper surface area of the specimen (mm^2^), L denotes the thickness of the specimen (mm), and H is the difference in the water level (mm).

#### 2.4.5. Porosity

**Figure 3:**
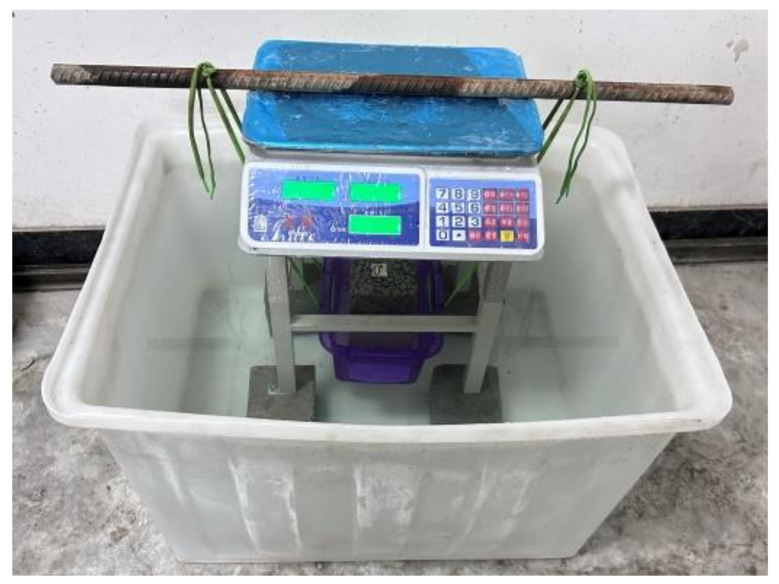
Porosity testing: homemade equipment.

The specimen was placed in a container with water, ensuring the water level was more than 20 cm higher than the specimen size. An electronic scale was used to measure the suspended mass of the specimen in water, denoted as $m_{1}$, when stability was achieved to record the mass. The submerged specimen was allowed to dry naturally until a constant weight was achieved, and the mass of the dry specimen was taken as $m_{2}$. The calculation formula is provided in Equation (5). The equipment employed for conducting the porosity test is depicted in Figure 3. The test for the porosity of the pervious concrete was carried out in accordance with the guidelines specified in CJJ/T135-2009, “Technical specifications for pervious concrete pavement” [35] and JCT2558-2020, “Pervious concrete” [36].

(5) $$P=(1-\frac{m_{2}-m_{1}}{\rho V})\times 100\%$$

where ρ is the density of water (g·cm^−3^), and V is the volume of the specimen.

## 3. Results and Analysis

### 3.1. Basic Performance Test Results

The outcomes of the tests for the cubic compressive strength, split tensile strength, flexural strength, permeability coefficient, and porosity in the pervious concrete for both the test group and the control group are presented in Table 6. Data from Table 5 reveal that the 28-day cubic compressive strength, splitting tensile strength, and flexural strength of the GBFS-HPMC/fiber pervious concrete attained maximum values of 25.22 MPa, 3.36 MPa, and 5.39 MPa, respectively. These values denote improvements of 25.29%, 11.26%, and 16.41%, respectively, when compared to the control group. Furthermore, within the test group, the permeability coefficient ranged from a maximum value of 6.23 mm·s^−1^ to a minimum value of 4.98 mm·s^−1^. Similarly, the maximum and minimum porosity values recorded were 14.62% and 13.65%, respectively. After the incorporation of GBFS, HPMC, and PPTF, a notable decrease in the permeability coefficient and porosity was observed. Despite this reduction, the values remained higher than those of the control group, which had a permeability coefficient of 6.35 mm·s^−1^ and a porosity of 14.89%. Nevertheless, all test groups met the stipulated specification requirements.

The results of the permeability coefficient, cubic compressive strength, and splitting tensile strength tests were subjected to ANOVA and extreme variance analysis using SPSS. The results are presented in Table 7 and Table 8, respectively. As evident from Table 8, the extreme variance effects of GBFS, HPMC, and PPTF on the mechanical and permeability properties of GBFS-HPMC/fiber pervious concrete are more significant than the effects of null columns, indicating the feasibility of the orthogonal test results. The standard deviations for cubic compressive strength, split tensile strength, flexural strength, water permeability coefficient, and porosity, calculated using SPSS, were 1.57, 0.1, 1.17, 0.35, and 0.4, respectively.

### 3.2. Mechanical Properties

#### 3.2.1. Compressive Strength of Cubes

**Figure 4:**
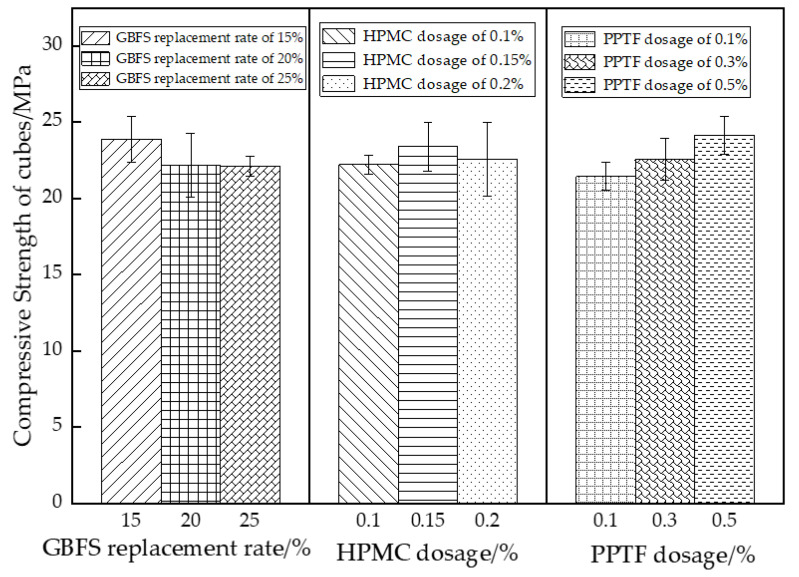
Effect of different factors on compressive strength of cubes.

The analysis of variance revealed that both GBFS and PPTF had a significant impact on the pervious concrete, whereas HPMC showed no significant effect. The magnitude of influence of the three factors on the cubic compressive strength was ranked by extreme variance analysis as follows: PPTF (2.89 MPa) > GBFS (1.75 MPa) > HPMC (0.52 MPa), consistent with the variance analysis results. The trend of the GBFS substitution rate, HPMC admixture, and PPTF fiber admixture effects on the cubic compressive strength of the GBFS-HPMC/fiber permeable concrete is illustrated in Figure 4.

As depicted in Figure 4, the cubic compressive strength of the GBFS-HPMC/fiber pervious concrete exhibited a decreasing trend with an increasing GBFS substitution rate. The cubic compressive strength decreased by 7.1% and 7.4%, respectively. GBFS possesses stronger hydration activity that consumes Ca(OH)_2_ generated by cement hydration and accelerates the cement hydration process [37,38]. This promotes the formation of C-S-H gel and improves the ITZ (aggregate–cement interfacial transition zone) of the matrix, resulting in a denser matrix structure and increased specimen strength. However, excessive GBFS addition prevents full gel hydration, causing tiny GBFS particles to only fill pores and reduce strength.

The cubic compressive strength increases initially with the increase in the HPMC dosage, reaching a maximum strength of 23.41 MPa at a dosage of 0.15%. During the mixing process, the -OH groups in HPMC react with water, forming hydrogen bonds. The molecules become entangled, creating a mesh structure [39]. This structure forms a gel-like film that envelops the aggregate, resulting in a denser structure. However, excessive HPMC addition increases the content of tiny air pores, subsequently reducing the specimen’s strength.

**Figure 5:**
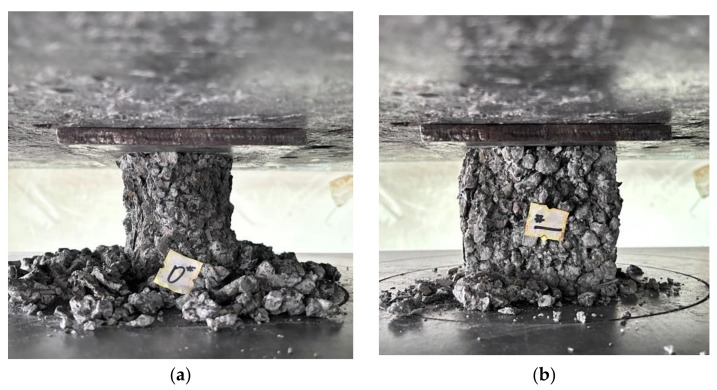

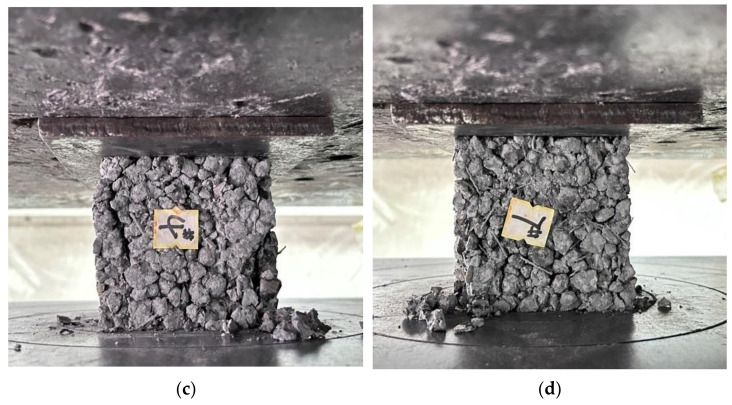
Cubic compressive damage pattern of specimen: (**a**) control group; (**b**) PPTF doping, 0.1; (**c**) PPTF doping, 0.3; (**d**) PPTF doping, 0.5.

The compressive strength gradually increased with the rise in the PPTF dosage, exhibiting increments of 6.51%, 12.22%, and 20.12% compared to the control group. The slope of the curve for an increase in the PPTF dosage from 0.3% to 0.5% was steeper than that for the increase in the fiber dosage from 0.1% to 0.3%. This observation confirms that a dosage of 0.5% PPTF significantly enhances the strength of pervious concrete. The fibers contribute to the overall densification of the pervious concrete and impede the propagation of cracks at the interface between the cementitious material and bonded aggregates [40]. The specimen’s load-bearing capacity increases with higher fiber admixture, considerably reducing aggregate shedding on the specimen’s surface. Figure 5 shows the specimen’s damage morphology.

#### 3.2.2. Splitting Tensile Strength

**Figure 6:**
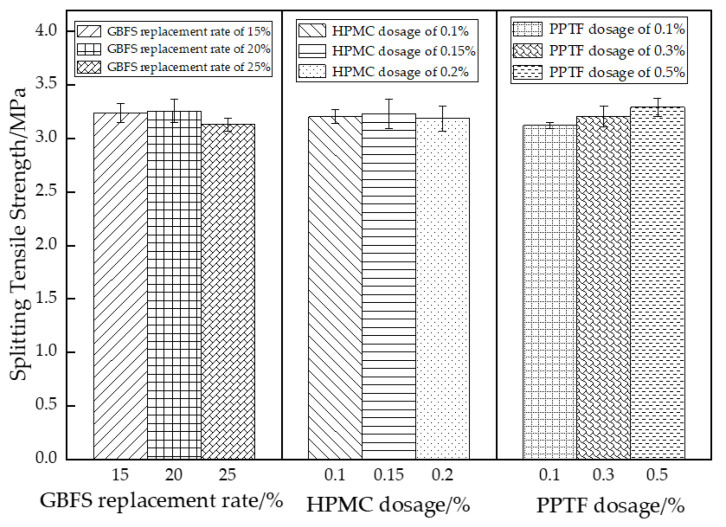
Effect of different factors on the splitting tensile strength.

The analysis of variance determined that PPTF is a significant factor, whereas GBFS and HPMC were found to be insignificant factors. Extreme variance analysis indicated the strength of influence on splitting tensile strength was PPTF (0.17 MPa) > GBFS (0.13 MPa) > HPMC (0.05 MPa). The trend of each factor’s influence on the splitting tensile strength is shown in Figure 6.

**Figure 7:**
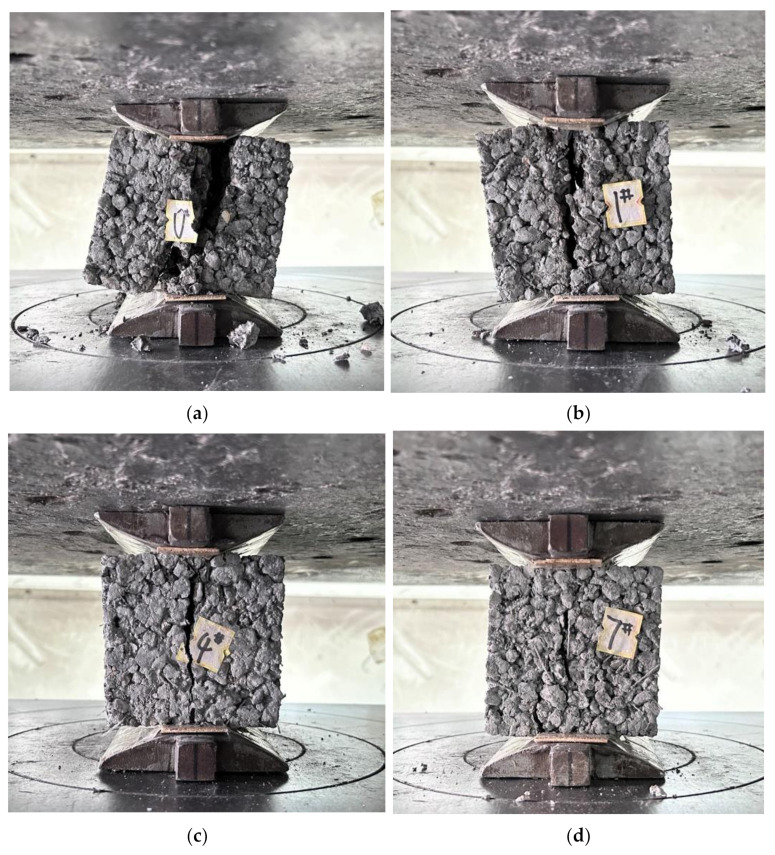
Split tensile damage pattern of specimen: (**a**) control group; (**b**) PPTF doping, 0.1; (**c**) PPTF doping, 0.3; (**d**) PPTF doping, 0.5.

As seen in Figure 6, the splitting tensile strength of the GBFS-HPMC/fiber pervious concrete increased and then decreased with a rising GBFS substitution rate. The highest splitting tensile strength of 3.26 MPa occurred at a 20% GBFS substitution rate. The increase in the splitting tensile strength was less pronounced as the mixing rate increased from 15% to 20%. The splitting tensile strength of the pervious concrete increased and then decreased with the increase in the HPMC admixture. This was mainly due to the fact that the addition of HPMC to the mix introduced tiny air bubbles, which improved the fluidity of the mix. However, excessive HPMC makes the specimens structurally loose, thereby reducing the splitting tensile strength of the specimens [41]. The addition of 0.5% PPTF fiber resulted in the highest splitting tensile strength. During the splitting process, the fiber can endure greater tensile stress and display an effective crack-resistant effect. Consequently, when the specimen undergoes splitting damage, it only forms penetrating cracks without complete fracture. The damage morphology is depicted in Figure 7.

#### 3.2.3. Flexural Strength

**Figure 8:**
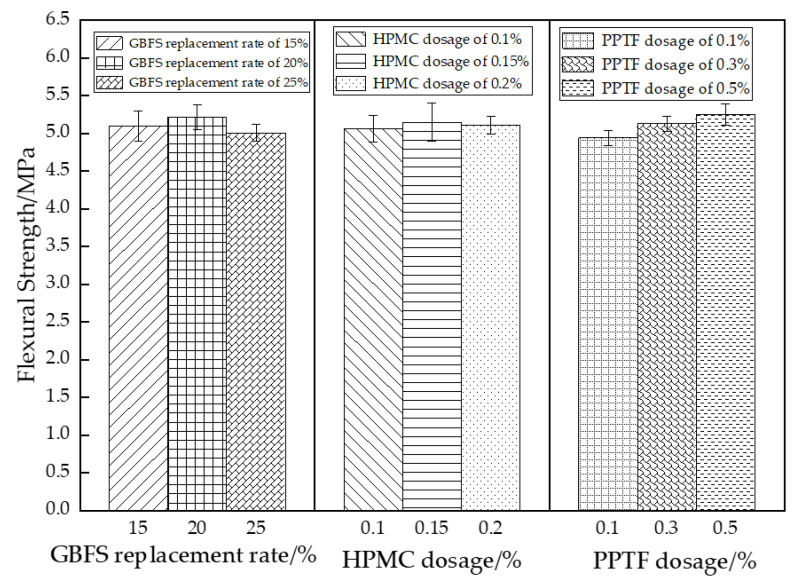
Effect of different factors on the flexural strength of thr pervious concrete.

The analysis of variance revealed both GBFS and PPTF as highly significant factors, while HPMC was found to be insignificant. The extreme variance analysis aligned with the variance results, demonstrating the strength of influence on the flexural strength as PPTF (0.31 MPa) > GBFS (0.21 MPa) > HPMC (0.09 MPa). The influence of each factor on flexural strength is illustrated in Figure 8.

**Figure 9:**
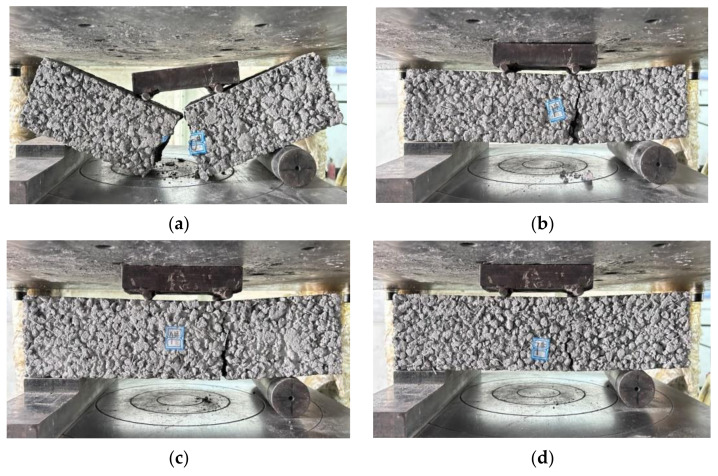
Flexural damage pattern of the specimen: (**a**) control group; (**b**) PPTF doping, 0.1; (**c**) PPTF doping, 0.3; (**d**) PPTF doping, 0.5.

As observed in Figure 8, the flexural strength of the GBFS-HPMC/fiber pervious concrete decreased as the GBFS replacement rate increased, peaking at a 15% GBFS replacement rate. The GBFS hydration product gel-encapsulated PPTF, enhancing the aggregate adhesion and bending moment capacity [42]. Nonetheless, an excessive amount of GBFS could not fully hydrate, leading to a decrease in the flexural strength. The highest flexural strength was achieved with a 0.15% HPMC dosage. The HPMC effectively distributed water evenly across the mortar surfaces, reducing the aggregate friction and thereby enhancing the flexural strength. However, excessive HPMC led to a reduced specimen densification, resulting in a weakening of the flexural strength. The flexural strength increased with the higher PPTF fiber doping due to the supporting and anchoring effect of the PPTF, which hindered the crack propagation when subjected to ultimate stress [43]. This damage morphology is depicted in Figure 9.

### 3.3. Permeability

**Figure 10:**
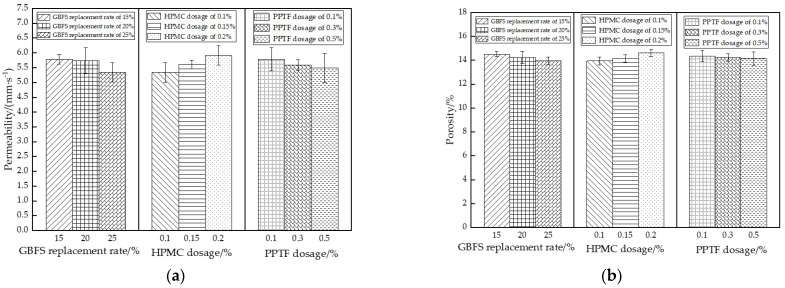
Influence of different factors on the permeability performance of pervious concrete: (**a**) water permeability coefficient; (**b**) porosity.

The analysis of variance revealed HPMC as a highly significant factor, GBFS as a significant factor, and PPTF as a nonsignificant factor in the permeability coefficient analysis. Similar results were obtained from the ANOVA based on porosity. The extreme variance analysis indicated the strength of influence on the permeability coefficient of the pervious concrete to be HPMC (0.58 mm/s) > GBFS (0.43 mm/s) > PPTF (0.28 mm/s). The influence on porosity followed the same order: HPMC (0.66%) > GBFS (0.57%) > PPTF (0.21%). The influence of each factor on the permeability coefficient and porosity is depicted in Figure 10.

As illustrated in Figure 10, a decline in the water permeability performance was observed as the rate of the increase in the GBFS substitution. This decline is attributed to the lower density of the GBFS. The material can be more effectively distributed within the cement paste, thereby filling internal pores within the sample [44]. However, when higher dosages of GBFS were employed, full hydration was not achieved. This incomplete hydration results in a reduction in the interconnecting pores in the pervious concrete, subsequently, leading to a decrease in its permeability coefficient. The addition of HPMC, on the other hand, enhanced the water permeability of the pervious concrete and improved the mixing conditions for its components. HPMC forms a membrane-like structure on the aggregate surface, increasing the water retention capacity of the mixture and reducing the occurrence of pore blockage to some extent. Furthermore, the permeability coefficient of pervious concrete decreases as the PPTF content increases. The surface of the fiber features wavy stripes that enhance its bond with the cement paste on the aggregate surface, resulting in a reduction of available pore space. The fibers intertwine to form a network that is dispersed within the pores of the pervious concrete.

**Figure 11:**
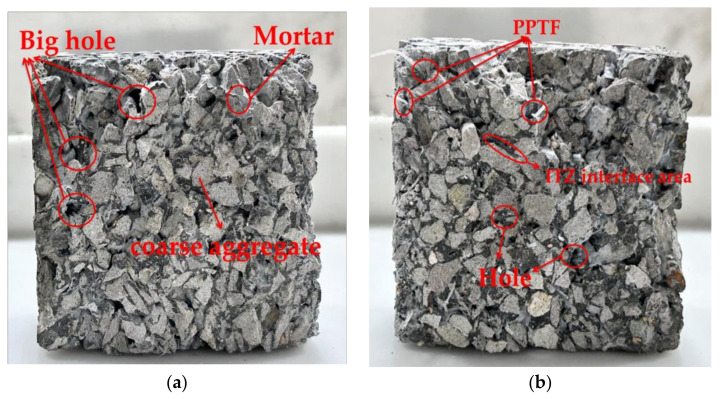
Specimen cross-section: (**a**) control group; (**b**) test group.

Figure 11 depicts both control and test groups of pervious concrete. GBFS and PPTF fill the pores within the concrete matrix in these groups. Consequently, the test group had fewer pores compared to the control group. 

## 4. Micromechanism Analysis

### 4.1. SEM Microanalysis

**Figure 12:**
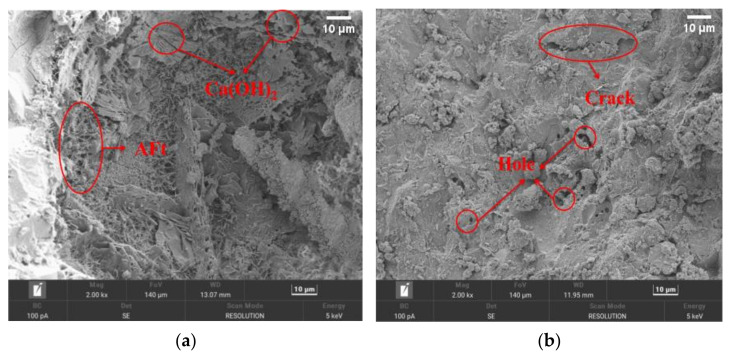

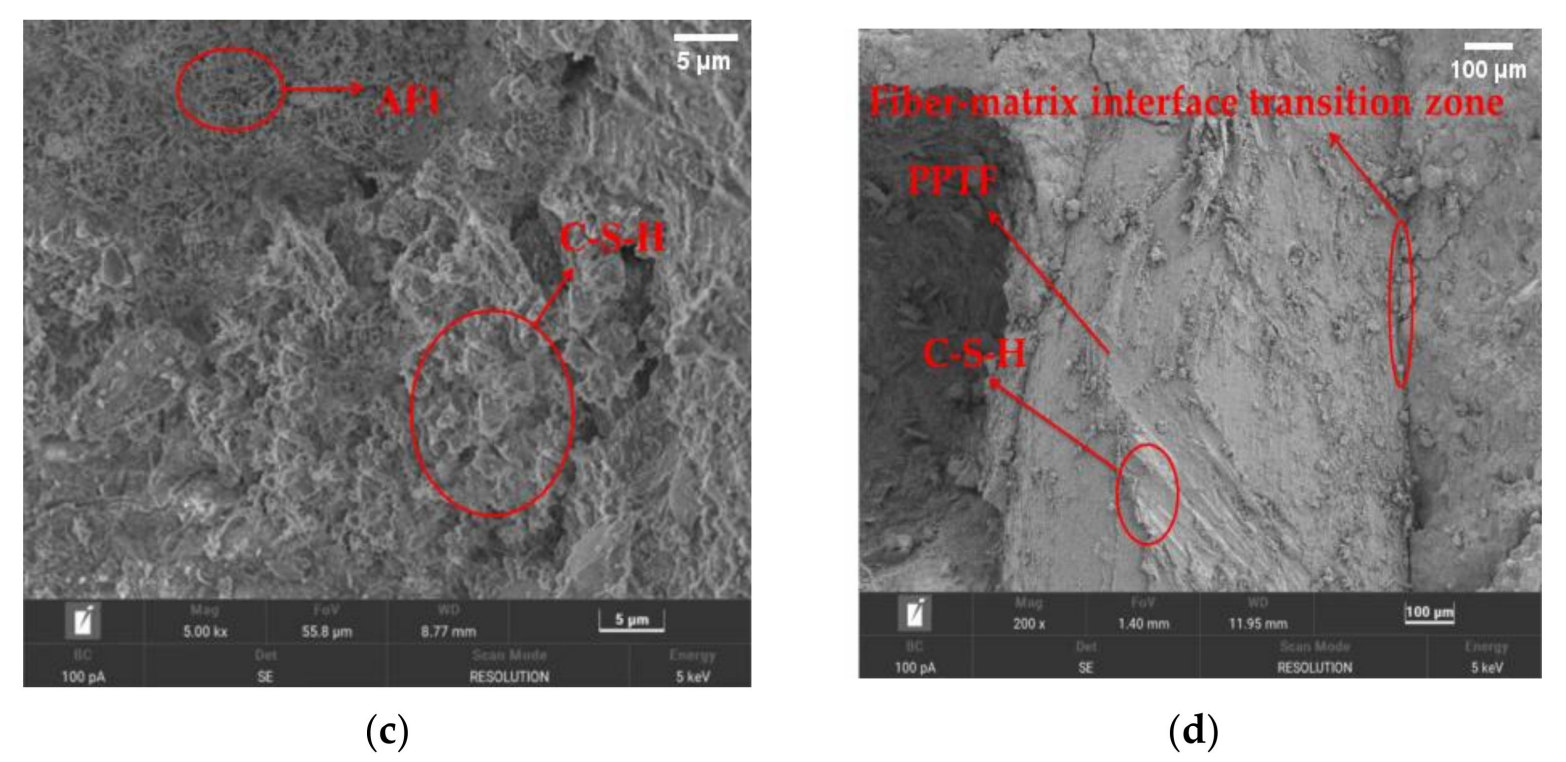
Micromorphological changes: (**a**) C-0—hydration products; (**b**) C-0—pore cracks; (**c**) GHP-3—hydration products; (**d**) GHP-3—fibers.

The micromorphologies of the C-0 and GHP-3 groups were observed through scanning electron microscopy (SEM), as depicted in Figure 12.

In the control group, the hydration products of the cement consisted of a small amount of acicular calcium alumina (AFt) and a large amount of flaky Ca(OH)_2_. The internal structure was not dense enough due to the usage of only cement as the cementitious material. This resulted in more holes and cracks, as shown in Figure 12a,b. The GBFS exhibits a volcanic ash effect. The Ca(OH)_2_, formed by the hydration of cement, along with the alkaline environment, leads to a secondary hydration reaction between GBFS and OH^−^. This resulted in the formation of part of AFt and AFm, as well as a substantial amount of C-S-H gel inside the permeable concrete. This gel reinforces the interface, resulting in a more compact structure that greatly improves the structural strength of permeable concrete, as shown in Figure 12b. The cured gel compounds encapsulate the fibers, strengthening the connection between the fibers and aggregates. Furthermore, the anchoring and supportive role of the fibers within the matrix restricts crack propagation, as depicted in Figure 12c.

### 4.2. EDS Energy Spectrum Analysis

**Figure 13:**
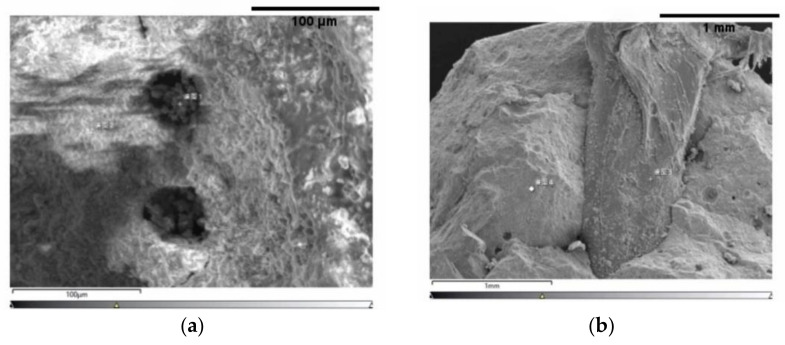
Energy spectrum hit location map: (**a**) C-0; (**b**) GHP-3.

**Figure 14:**
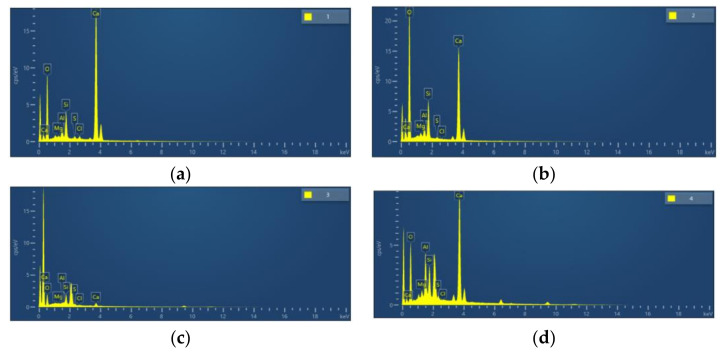
Specimen energy spectrum: (**a**) energy spectrum of region 1; (**b**) energy spectrum of region 2; (**c**) energy spectrum of region 3; (**d**) energy spectrum of region 4.

The EDS energy spectrum analysis located hit points and depicted energy spectra, as shown in Figure 13 and Figure 14. Chemical elemental analysis yielded the sample’s composition, as detailed in Table 9.

Hydration products in the control group’s constituent elements in both regions were Ca, O, and S. The O and Ca elements were predominant, with CaO, MgO, etc., indicating the presence of Ca(OH)_2_, AFt, and some AFm. GHP-3’s hydration product constituent elements in both regions were Ca, O, Al, and Si. The Si, Al, and Mg levels were higher than in C-0, with CaO, Al2O3, SiO2, and MgO indicating the presence of Ca(OH)_2_, AFt, and some AFm. This suggests the existence of AFt and a significant amount of C-S-H gel in this region.

## 5. Doping Optimization

### 5.1. Gray Correlation Analysis

The design of the orthogonal experiments effectively reduces the number of experimental groups, thereby significantly reducing the workload. Nevertheless, the results of the remaining experimental data remain unknown. Gray correlation analysis provides a quantitative method to describe and predict performance indicators across the entire system [45]. The correlation among variables is calculated by employing data from gray correlation to obtain the best experimental results [46]. Principal component analysis transforms correlated variables into linearly uncorrelated variables through orthogonal transformation. This process determines the weights of each factor by establishing coefficients of principal components in each linear combination and normalizing them.

The calculation of the gray correlation coefficient is shown in Equation (6):

(6) $$\zeta_{m}(k)=\frac{\underset{m}{\min\;}\underset{k}{\min}\left|x_{0}(k)-f(x_{m}(k))\right|+\rho \cdot \underset{m}{\max\;}\underset{k}{\max}\left|x_{0}(k)-f(x_{m}(k))\right|}{\left|x_{0}(k)-f(x_{m}(k))\right|+\rho \cdot \underset{m}{\max\;}\underset{k}{\max}\left|x_{0}(k)-f(x_{m}(k))\right|}$$

where $f(x_{m}(k))=\frac{x_{m}(k)-\underset{k}{\min}\;x_{m}(k)}{\underset{k}{\max}\;x_{m}(k)-\underset{k}{\min}\;x_{m}(k)}$, $f(x_{m}(k))$ are the dimensionless values of the kth property of the mth group of ratios; $x_{m}(k)$, $\underset{k}{\max}\;x_{m}(k)$, and $\underset{k}{\min}\;x_{m}(k)$ are the measured, maximum, and minimum values of the kth property of the mth group of ratios; and ρ is the resolution factor, which is usually taken as 0.5.

Next, the performance indicators are subjected to SPSS factor analysis. The coefficients of each factor in the composite score model are normalized to obtain the weights. The weight calculation is shown in Equation (7):

(7) $$\eta_{m}=\sum_{k=1}^{n}\omega_{k}\zeta_{mk}$$

where $\omega_{k}$ is the weighting factor of the k-th key figure, and n is the number of key figures.

### 5.2. Gray Correlation Coefficients and Weighting Factors

Gray correlation analysis [47] and principal component analysis were utilized to determine the optimal admixture of the GBFS-HPMC/fiber pervious concrete. The five performance indicators, namely, cubic compressive strength, splitting tensile strength, flexural strength, permeability coefficient, and porosity (listed in Table 6), were substituted into Equation (6) to calculate the gray correlation coefficients of the GBFS-HPMC/fiber pervious concrete, as shown in Table 10. These performance indicators were also fed into SPSS (IBM SPSS Statistics 26) software to obtain the total variance explained and the component matrix through dimensionality reduction processing, presented in Table 11 and Table 12, respectively.

The weighting coefficients for the cubic compressive strength, splitting tensile strength, flexural strength, water permeability, and porosity were calculated as $\omega_{1}$ = 0.2084, $\omega_{2}$ = 0.1519, $\omega_{3}$ = 0.1885, $\omega_{4}$ = 0.2111, and $\omega_{5}$ = 0.2401, respectively. The gray correlation coefficients of each experimental group were weighted using Equation (2), resulting in gray correlations for each group, as presented in Table 13.

Gray correlation measures the strength of the correlation between different influencing factors and reference objects. A higher gray correlation indicates a stronger alignment between the trends in test results and the ideal experimental outcomes. This implies better overall performance within the experimental group. Based on these considerations, GHP-3 is determined to be the best experimental scheme for comprehensive performance indexes.

### 5.3. Doping Optimization

The dosage combination yielding the best combined performance index was analyzed by comparing the mean gray correlation for each dosage of every factor, as shown in Table 14.

The GHP-10 test group, consisting of GBFS-HPMC/fiber pervious concrete with the optimal GBFS replacement rate of 20%, HPMC admixture of 0.20%, and PPTF admixture of 0.5% was chosen to evaluate the comprehensive performance indexes. New pervious concrete specimens were prepared using the best admixture determined through gray correlation analysis for both the GHP-10 and GHP-3 test groups. These specimens were then measured for five performance indices: cubic compressive strength, tensile strength, flexural strength, water permeability coefficient, and porosity. The final optimal admixture formulation was derived from these measurements. The data from both sets of programs are presented in Table 15. Upon comparing the two datasets, it was observed that the cubic compressive strength, splitting tensile strength, and flexural strength of GBFS-HPMC/fiber pervious concrete increased by 7.2%, 2.1%, and 2.5%, respectively. In contrast, the water permeability coefficient decreased by 5.5%, and porosity decreased by 3.6%. The optimized solution’s water permeability coefficient met the specified requirements, resulting in improved mechanical properties.

## 6. Conclusions

This experiment explored the effects of GBFS, HPMC, and PPTF on pervious concrete’s mechanical properties from macrostructural and micromorphological perspectives. The 28-day cubic compressive strength, splitting tensile strength, and flexural strength of GBFS-HPMC/fiber pervious concrete reached the highest values of 25.22 MPa, 3.36 MPa, and 5.39 MPa, respectively. These values exceeded those of the control group by 25.29%, 11.26%, and 16.41%, respectively. Furthermore, the water permeability coefficient and porosity met specifications.

The analysis of variance on mechanical properties revealed that PPTF exerted the most substantial influence, followed by GBFS, whereas HPMC had negligible effects. Conversely, the analysis of variance regarding water permeability properties emphasized HPMC as a significant influencing factor, while GBFS and PPTF had minor effects. This observation aligns with the results obtained from the extreme variance analysis. Based on comprehensive analysis, the optimal dosages for GBFS, HPMC, and PPTF to enhance compressive strength are 15%, 0.15%, and 0.5%, respectively. For split tensile strength and flexural strength, the recommended dosages of GBFS, HPMC, and PPTF are 20%, 0.15%, and 0.5%, respectively. To enhance water permeability, the recommended dosages of GBFS, HPMC, and PPTF are 15%, 0.2%, and 0.1%, respectively. SEM microstructure analysis has demonstrated that incorporating GBFS enhances the stability and compactness of the pervious concrete structure. This enhancement is attributed to the secondary hydration of GBFS, which results in the formation of C-S-H gel. This gel fills the pores and reinforces the fiber–aggregate interfacial transition zone. Energy spectroscopy analysis validated the effectiveness of the fibers by detecting the presence of AFt and a substantial amount of C-S-H gel in the GHP-3 test group.

Finally, through gray correlation analysis and SPSS factor dimensionality reduction for admixture optimization, we obtained the optimal admixture proportions for GBFS-HPMC/fiber permeable concrete: a GBFS replacement rate of 20%, an HPMC volume admixture of 0.20%, and a fiber volume admixture of 0.5%. As a consequence of this optimization, the GBFS-HPMC/fiber pervious concrete exhibited a 7.2% increase in cubic compressive strength, a 2.1% increase in split tensile strength, and a 2.5% increase in flexural strength. Additionally, the permeability coefficient decreased by 5.5%, and porosity decreased by 3.6%. Notably, the optimized water permeability still complies with the specified requirements, thereby enhancing the material’s mechanical properties. These findings establish a theoretical foundation for the promotion and application of pervious concrete.

## Figures and Tables

**Table 1:** Physical properties of cement.

Cement Varieties	Densities/(g/cm^3^)	Initial Setting Time/min	Final Setting Time/min	Flexural Strength/MPa		Compressive Strength /MPa	
				3d	28d	3d	28d
P·O42.5	3.1	160.5	260.5	5.18	8.88	24.59	49.87

**Table 2:** Composition and properties of GBFS.

Fluidity Ratio/%	Activity Index/%		Densities/(g/cm^3^)	Loss on Ignition/%	Water Content/%	Composition and Properties/(wt%)					
	7d	28d				CaO	SiO_2_	Al_2_O_3_	Fe_2_O_3_	SO_3_	MgO
98.00	84.20	98.50	3.10	0.84	0.45	34.00	34.50	17.70	1.03	1.64	6.01

**Table 3:** Technical specifications of plastisol fiber.

Primary Material	Nominal Length/mm	Density /(g/cm^3^)	Equivalent Diameter/mm	Breaking Strength/MPa	Elongation at Break/%	Initial Modulus/GPa
Modified polypropylene resins	20	0.91	>0.1	≥450	≤30	≥5

**Table 4:** Table of factor levels for the orthogonal experimental design.

Level	Factor			
	GBFS (A)/%	HPMC (B)/%	PPTF (C)/%	Blank Column
1	15	0.1	0.1	1
2	20	0.15	0.3	2
3	25	0.2	0.5	3

**Table 5:** Pervious concrete mixing ratios.

Group	Crushed Stone	Sand	Water	Cement	Slag	Cellulose	Plastic Fiber
C-0	1424.43	74.97	134.75	481.26	0	0	0
GHP-1	1424.43	74.97	134.75	409.07	72.19	0.48	0.91
GHP-2	1424.43	74.97	134.75	409.07	72.19	0.72	2.73
GHP-3	1424.43	74.97	134.75	409.07	72.19	0.96	4.55
GHP-4	1424.43	74.97	134.75	385.01	96.25	0.48	2.73
GHP-5	1424.43	74.97	134.75	385.01	96.25	0.72	4.55
GHP-6	1424.43	74.97	134.75	385.01	96.25	0.96	0.91
GHP-7	1424.43	74.97	134.75	360.95	120.32	0.48	4.55
GHP-8	1424.43	74.97	134.75	360.95	120.32	0.72	0.91
GHP-9	1424.43	74.97	134.75	360.95	120.32	0.96	2.73

**Table 6:** Test results of mechanical properties and water permeability of the pervious concrete.

Group	GBFS /vol%	HPMC/wt%	PPTF /vol%	Cubic Compressive Strength/MPa	Splitting Tensile Strength/MPa	Flexural Strength/MPa	Permeability/(mm·s^−1^)	Porosity/%
C-0	0	0	0	20.13	3.02	4.63	6.35	15.08
GHP-1	15	0.1	0.1	22.29	3.14	4.87	5.63	14.31
GHP-2	15	0.15	0.3	24.17	3.25	5.17	5.77	14.51
GHP-3	15	0.2	0.5	25.22	3.32	5.25	5.95	14.75
GHP-4	20	0.1	0.3	21.60	3.27	5.21	5.41	13.88
GHP-5	20	0.15	0.5	24.50	3.36	5.39	5.56	14.02
GHP-6	20	0.2	0.1	20.48	3.14	5.06	6.23	14.82
GHP-7	25	0.1	0.5	22.81	3.20	5.11	4.98	13.65
GHP-8	25	0.15	0.1	21.56	3.09	4.89	5.49	13.93
GHP-9	25	0.2	0.3	22.01	3.10	5.02	5.58	14.27

C is the control group; GHP is the GBFS-HPMC/fiber pervious concrete.

**Table 7:** ANOVA for mechanical and water permeability properties.

Performance Indicators	Source of Variation	Ss	Df	Ms	F	P
Cubic compressive strength (f_cu_)/MPa	GBFS	6.016	2	3.008	19.209	0.049
	HPMC	2.203	2	1.102	7.036	0.124
	PPTF	11.301	2	5.650	36.086	0.027
	Error	0.313	2	0.157		
Splitting tensile strength (f_ts_)/MPa	GBFS	0.028	2	0.014	17.151	0.055
	HPMC	0.003	2	0.002	2.068	0.326
	PPTF	0.043	2	0.022	26.726	0.036
	Error	0.002	2	0.001		
Flexural strength (f_f_)/MPa	GBFS	0.069	2	0.034	72.023	0.014
	HPMC	0.011	2	0.006	11.814	0.078
	PPTF	0.147	2	0.074	153.930	0.006
	Error	0.001	2	0.000		
Permeability/(mm·s^−1^)	GBFS	0.337	2	0.169	45.030	0.022
	HPMC	0.506	2	0.253	67.525	0.015
	PPTF	0.129	2	0.064	17.220	0.055
	Error	0.007	2	0.004		
Porosity/%	GBFS	4.931 × 10^−5^	2	2.465 × 10^−5^	13.765	0.068
	HPMC	6.988 × 10^−5^	2	3.494 × 10^−5^	19.506	0.049
	PPTF	6.969 × 10^−6^	2	3.484 × 10^−6^	1.945	0.340
	Error	3.582 × 10^−6^	2	1.791 × 10^−6^		

For this test, a significance level of 0.05 was set, and the degrees of freedom were (2,2) to determine the distribution critical value of F0. The value of 0.05 (2,2) is 19.00.

**Table 8:** Polar analysis of the mechanical and water permeability properties.

Performance Indicators	Calculation Range	GBFS	HPMC	PPTF	Blank Column
Cubic compressive strength (f_cu_)/MPa	K1j	23.89	22.23	21.44	22.93
	K2j	22.19	23.41	22.59	22.49
	K3j	22.13	22.57	24.18	22.79
	Rj	1.76	1.18	2.74	0.44
Splitting tensile strength (f_ts_)/MPa	K1j	3.24	3.20	3.12	3.20
	K2j	3.26	3.23	3.21	3.20
	K3j	3.13	3.19	3.29	3.23
	Rj	0.13	0.04	0.17	0.03
Flexural strength (f_f_)/MPa	K1j	5.1	5.06	4.94	5.09
	K2j	5.22	5.15	5.13	5.11
	K3j	5.01	5.11	5.25	5.12
	Rj	0.21	0.09	0.31	0.03
Permeability/(mm·s^−1^)	K1j	5.78	5.34	5.78	5.59
	K2j	5.73	5.61	5.59	5.66
	K3j	5.35	5.92	5.50	5.62
	Rj	0.43	0.58	0.28	0.07
Porosity/%	K1j	14.52	13.95	14.35	14.20
	K2j	14.24	14.15	14.22	14.33
	K3j	13.95	14.61	14.14	14.19
	Rj	0.57	0.66	0.21	0.13

**Table 9:** Elemental composition of the test pieces.

Elemental	Spectrogram 1	Spectrogram 2	Spectrogram 3	Spectrogram 4
O	51.09	65.11	64.00	47.13
Mg	0.27	0.60	1.21	1.38
Al	1.59	1.71	0.40	7.30
Si	4.06	5.27	21.21	6.00
S	0.39	0.24	0.08	0.19
Cl	0.56	0.05	1.14	0.11
Ca	42.03	27.03	12.43	37.88
Total	100.00	100.00	100.00	100.00

**Table 10:** Gray correlation coefficients.

Test Number	Performance Indicators				
	Cubic Compressive Strength	Splitting Tensile Strength	Flexural Strength	Permeability	Porosity
GHP-1	0.4472	0.3803	0.3333	0.5102	0.3333
GHP-2	0.6930	0.5510	0.5909	0.5760	0.4513
GHP-3	1.0000	0.7714	0.7222	0.6906	0.7846
GHP-4	0.3957	0.6000	0.6500	0.4325	0.2134
GHP-5	0.7670	1.0000	1.0000	0.4826	0.2417
GHP-6	0.3333	0.3803	0.4727	1.0000	1.0000
GHP-7	0.4958	0.4576	0.5200	0.3333	0.1789
GHP-8	0.3930	0.3333	0.3611	0.4579	0.2227
GHP-9	0.4247	0.3418	0.4407	0.4902	0.3168

**Table 11:** Total variance explained.

Ingredient	Eigenvalue λ	Contribution Rate/%	Cumulative Contribution Rate/%
1	2.625	52.508	52.508
2	1.979	39.585	92.093
3	0.364	7.282	99.374
4	0.02	0.407	99.781
5	0.011	0.219	100

**Table 12:** Component matrix.

Performance Indicators	Ingredient	
	1	2
Cubic compressive strength	0.871	0.088
Splitting tensile strength	0.979	−0.102
Flexural strength	0.948	−0.094
Permeability	0.014	0.986
Porosity	0.099	0.99

**Table 13:** Gray correlation.

Group	Factor Admixture			Gray Correlation
	GBFS/%	HPMC/%	PPTF/%	
GHP-1	15	0.1	0.1	0.4003
GHP-2	15	0.15	0.3	0.5720
GHP-3	15	0.2	0.5	0.7971
GHP-4	20	0.1	0.3	0.4529
GHP-5	20	0.15	0.5	0.6916
GHP-6	20	0.2	0.1	0.6382
GHP-7	25	0.1	0.5	0.3947
GHP-8	25	0.15	0.1	0.3516
GHP-9	25	0.2	0.3	0.4012

**Table 14:** Average gray correlation.

Considerations	Substitution Rate or Dosage	Average Gray Correlation
GBFS/%	15	0.5898
	20	0.5942
	25	0.3825
HPMC/%	0.1	0.4160
	0.15	0.5384
	0.2	0.6122
PPTF/%	0.1	0.4634
	0.3	0.4754
	0.5	0.6278

**Table 15:** Performance optimization metrics.

Performance Indicators	GHP-3 Group	GHP-10 Group	Rate of Change/%
Cubic compressive strength/MPa	25.22	27.03	7.2
Splitting tensile strength/MPa	3.32	3.39	2.1
Flexural strength/MPa	5.23	5.36	2.5
Permeability/(mm·s^−1^)	5.95	5.62	−5.5
Porosity/%	14.75	14.22	−3.6

## Data Availability

Not applicable.

## Abbreviations

GBFS: Granulated Blast-Furnace Slag; HPMC: Hydroxypropyl Methylcellulose; PPTF: Polypropylene Plastic Textile Fiber.
